# Arrhythmia as a Possible Complication of Mycophenolate Mofetil in Systemic Sclerosis: A Case Report

**DOI:** 10.1155/carm/8858671

**Published:** 2025-02-12

**Authors:** Zahra Moradi, Vahid Ardestani, Zahra Tamartash, Elaheh Karimi, Hoda Kavosi

**Affiliations:** ^1^School of Medicine, Tehran University of Medical Sciences, Tehran, Iran; ^2^Rheumatology Research Center, Tehran University of Medical Sciences, Tehran, Iran

**Keywords:** arrhythmia, CellCept, mycophenolate mofetil, systemic sclerosis

## Abstract

A 55-year-old male patient with diffuse cutaneous systemic sclerosis (dcSSC) since 2018 presented with a history of arrhythmia. He had been stable for 5 years with pantoprazole, diltiazem, and mycophenolate mofetil (MMF); vitamin E; and vitamin D until he developed arrhythmia. Different evaluations revealed left bundle branch block, wall motion abnormality, mildly reduced systolic function, diffused interstitial fibrosis, and lesions in the left circumflex artery (LCX) and left anterior descending artery (LAD) and stenosis in LCX, without significant improvement following percutaneous coronary intervention for LCX stenosis. Holter monitoring demonstrated persistent ventricular premature beats and couplets. Arrhythmia was not responsive to bisoprolol therapy, and it was not feasible to perform cardiac ablation. Suspecting MMF–induced arrhythmia, MMF was discontinued, which led to a reduction in arrhythmia and symptom improvement after 9 months. This case report emphasized a possible heart-related complication of MMF, which healthcare providers should consider when prescribing medication to patients.

## 1. Introduction

Systemic sclerosis (SSC) is defined by immune dysfunction, vasculopathy, cellular inflammation, and fibrosis of the skin and multiple internal organs [1]. Among internal organ involvement, interstitial lung disease (ILD) and cardiac involvement are associated with poor outcomes and continue to be the main leading cause of death, responsible for 23% and 29% of overall mortality in SSc patients, respectively [2].

Mycophenolate mofetil (MMF), as an immunosuppressant agent, is frequently used in systemic sclerosis–related skin diseases and patients with ILD [3]. Despite the safety of MMF, several side effects have been reported, which include gastrointestinal disturbance, myelosuppression, and increased risk of infection [4]. In addition, the Food and Drug Administration (FDA) suggested arrhythmia as a cardiac complication related to MMF use in combination with cyclosporine and corticosteroids [5]. This case report noted the potential relationship between MMF administration and arrhythmia.

## 2. Case Presentation

**Figure 1:**
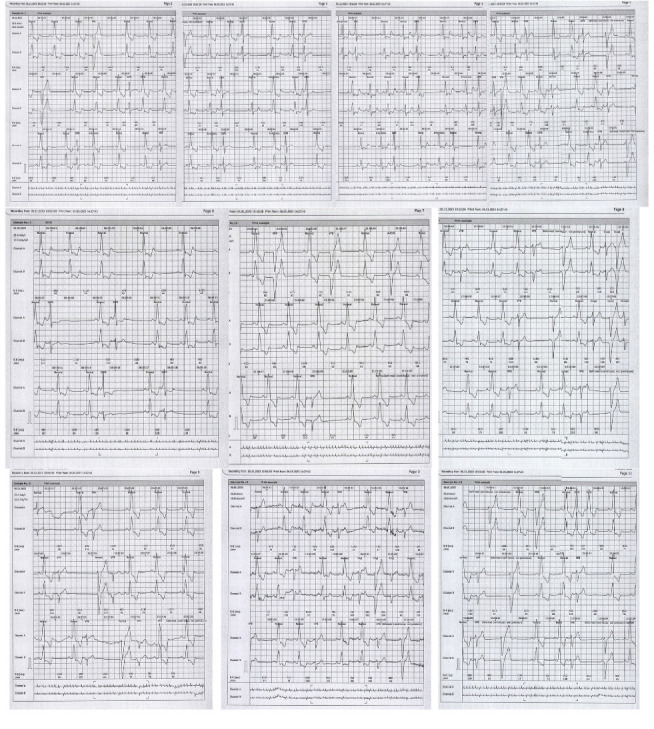
The first Holter revealed frequent ventricular ectopies with different morphology and bundle branch blocks throughout the recording period.

**Figure 2:**
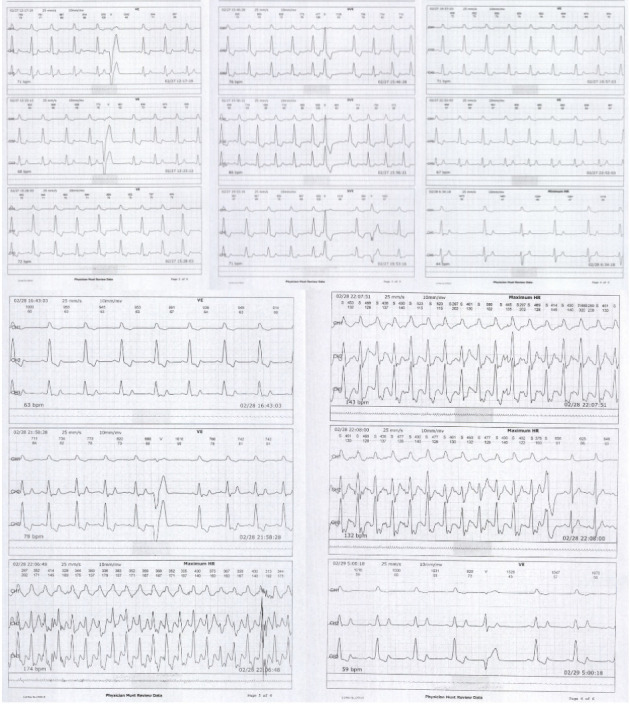
The third Holter revealed infrequent ventricular and atrial ectopies, and one episode of supraventricular tachycardia, which decreased compared to the findings of the first Holter.

A 55-year-old man with diffuse cutaneous SSC (dcSSC) since 2018 presented to our clinic for the evaluation of arrhythmia. The diagnosis was based on extensive skin involvement, a modified Rodnan skin score of 29, the presence of Raynaud's phenomenon, and a capillaroscopy of late SSC. He did not have lung involvement since high-resolution computed tomography and pulmonary function test (PFT) were normal (forced vital capacity of 93% predicted and carbon monoxide diffusion of 93% predicted). Echocardiography also revealed Grade I left ventricular diastolic dysfunction, an ejection fraction (EF) of 55%, a pulmonary artery pressure (PAP) of 14 mmHg, a tricuspid regurgitant jet of 15, trivial mitral regurgitation (MR), and trivial tricuspid valve regurgitation (TR). Lab data demonstrated positive antinuclear antibody, negative anti-centromere antibody, and negative anti-topoisomerase I (Scl-70) antibody. His drug history included pantoprazole 20 mg daily, diltiazem 120 mg daily, MMF 2 gr daily, vitamin E, and vitamin D. He was stable for 5 years until he developed frequent PVCs. In addition, the electrocardiogram demonstrated a left bundle branch block. Echocardiography was performed for the patient, and it showed an EF of 50%, a PAP of 29 mmHg, mild to moderate MR, mild TR, and wall motion abnormality. Cardiac magnetic resonance imaging findings also demonstrated mildly enlarged left ventricle size without left ventricular hypertrophy, mildly reduced systolic function, abnormal septal motion due to left bundle branch block, increased global T2, diffused interstitial fibrosis, increased global T1 values without late gadolinium enhancement, and functional assessments, which were compatible with long-term cardiac involvement. Myocardial perfusion single-photon emission computed tomography (SPECT) also reported decreased radiotracer uptake in anteroseptal with partially better uptake in the rest phase, suggestive of mild ischemia of the anterolateral wall. Based on imaging results, angiography was performed, which revealed lesions in the left circumflex artery (LCX) and left anterior descending artery (LAD) and 80% stenosis in LCX, resulting in percutaneous coronary intervention on LCX. However, no improvement in arrhythmia was achieved. In addition, a Holter was placed. The result of the first Holter monitor demonstrated 78,886 ventricular premature beats, 9794 ventricular couplet episodes, 19,588 QRS couplets, and bundle branch blocks throughout the recording (Figure 1). Due to diffuse arrhythmia, it was not able to perform cardiac ablation, so the patient was prescribed bisoprolol 2.5 mg daily. The second Holter was performed after using a beta-blocker for 3 months, which did not significantly improve the patient's arrhythmia (Supporting Figure 1). Considering the persistence of arrhythmia, the cardiologist hypothesized that arrhythmia occurred due to MMF use. So, we decided to discontinue MMF. After 9 months, the patient's arrhythmia improved since the result of the third Holter reported 3917 ventricular ectopies and 177 supraventricular ectopies, and the patient's symptoms were relieved (Figure 2).

## 3. Discussion

SSC can affect internal organs and is known for its frequent and severe cardiovascular complications. SSC can affect all three parts of the heart such as myocardium, pericardium, and endocardium [6]. Myocardial involvement can manifest as focal ischemia, recurrent ischemia–reperfusion injury, myocarditis, and myocardial fibrosis. Pericardial involvement includes fibrinous pericarditis, pericardial adhesions, pericardial effusion, pericardial tamponade, and constrictive pericarditis. In addition, endocardial involvement may result in valvular vegetation and endocarditis as an extremely rare event [6]. Arrhythmia is a notable presentation of cardiac involvement in SSC, linked to unfavorable outcomes and 6% of deaths in the extensive European League Against Rheumatism (EULAR) Scleroderma Trials and Research (EUSTAR) database [7].

MMF is widely used as a preferred option for treating skin disease associated with SSC, particularly in patients with ILD or progressive skin disease who cannot tolerate methotrexate [3]. MMF, however, has been associated with several potential side effects, such as gastrointestinal symptoms and bone marrow suppression, as well as other complications, including hepatitis, an increased risk of infection, cancer, and progressive multifocal leukoencephalopathy, some of which are dose-dependent [4].

To date, there has been limited research on the potential cardiac complications of using MMF. The relationship between MMF and cardiac events, such as angina, arrhythmia, arterial thrombosis, pericardial effusion, congestive heart failure, hypertension, hypotension, tachycardia, lower extremity edema, phlebitis, endocarditis, and venous thrombosis, is uncertain but has been highlighted by the FDA [5]. However, clinical studies reporting these complications are rare. Becker et al. showed that the administration of TAC/MMF in patients with liver transplants would be associated with the occurrence of supraventricular arrhythmia, as seen in 2.7% of patients [8]. Also, in another study by Eisen et al., a low rate of atrial arrhythmia is reported in cardiac transplant recipients after 3 years of using MMF [9].

The study presented a patient with SSC who developed arrhythmia several years after diagnosis. Arrhythmia in this patient may result from various factors, including cardiac involvement in SSC. In SSC, microvascular dysfunction leads to patchy myocardial fibrosis and contraction band necrosis. Over time, progressive replacement fibrosis affects both ventricles and the conduction system. This disruption in electrical pathways can facilitate the formation of reentry circuits. In addition, autonomic dysfunction in SSC further increases the risk of arrhythmias [10]. Other causes include drug complications, electrolyte imbalances, and drug interactions. Initially, the arrhythmia was attributed to known cardiac complications of SSC, and antiarrhythmic medication was prescribed. However, the persistence of symptoms despite treatment led to MMF being suspected as a contributing factor. In collaboration with a cardiologist, MMF was discontinued for a while and the Holter results showed a reduction in arrhythmia severity, supporting the hypothesis that MMF may have played a role in the exacerbation of cardiac complications. The exact mechanism of MMF causing cardiac arrhythmias is unclear, but one possible explanation is that the immunosuppressive effects of purine synthesis inhibitors such as MMF may affect myocardial structural remodeling, potentially leading to decreased conduction velocity or altered refractory periods. Furthermore, it is possible that the agent could cause damage to the cardiac conduction system and induce toxic immunogenic reactions to cardiomyocytes [11, 12]. Since after 9 months of follow-up, the patients still had ectopic beats despite the significant decrease in severity, it can be assumed that MMF exacerbated the arrhythmia that was previously developed as a cardiac complication of SSC. Further studies are needed to elucidate the relationship between MMF and cardiac events in SSC and determine the exact underlying mechanisms related to cardiac complications.

In conclusion, as cardiac involvement and arrhythmias are well-documented manifestations of SSC, itself, our case highlights an important consideration regarding the use of immunosuppressive medications such as MMF. Although MMF remains a valuable therapeutic option for managing underlying SSC manifestations, particularly in cases with organ involvement, clinicians should be aware that it may potentially exacerbate cardiac complications, especially arrhythmias, during long-term use. The temporal relationship between MMF discontinuation and arrhythmia improvement in our patient suggests a possible causative or aggravating role of the medication. This case emphasizes the importance of careful cardiac monitoring in SSC patients receiving MMF, particularly during extended treatment periods.

## Data Availability

Data sharing is not applicable to this article as no new data were created or analyzed in this study.

## Nomenclature

EF: Ejection fraction
ILD: Interstitial lung disease
LCX: Left circumflex artery
MMF: Mycophenolate mofetil
MR: Mitral regurgitation
TR: Tricuspid regurgitation
PAP: Pulmonary artery pressure
PFT: Pulmonary function test
SSC: Systemic sclerosis

## Supporting Information

The supporting information for this article includes one figure related to the second Holter monitor analysis.
