# Supplementary material for: Arrhythmia as a Possible Complication of Mycophenolate Mofetil in Systemic Sclerosis: A Case Report
Source: Case Rep Med. 2025 Feb 12;2025:8858671. doi: 10.1155/carm/8858671 (PMC11839254; doi:10.1155/carm/8858671)
Supplement: Supporting Information — Additional supporting information can be found online in the Supporting Information section. [file 8858671.f1.docx]

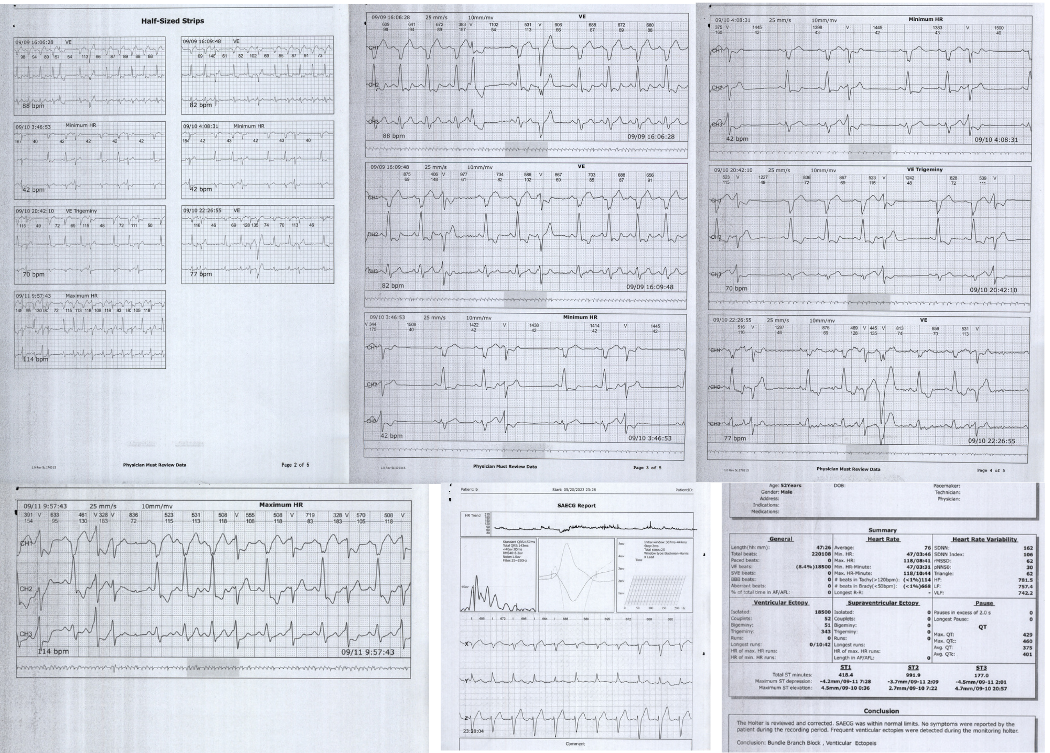


**Fig S1.** The second Holter demonstrated 18946 ventricular ectopies (18500 isolated ectopies, 52 couplets, 51 bigeminy ectopies), which did not show significant improvement compared to the first Holter.
